# Yogurt Fortification With Lyophilized Liposomes Coencapsulating Vitamins D_3_ and B_12_: Physicochemical Characterization, Sensory Evaluation and Static In Vitro Digestion

**DOI:** 10.1111/1750-3841.70870

**Published:** 2026-02-06

**Authors:** Letícia S. Ferreira, Eduarda H. Luvizzotti, Marluci Ghiraldi, Matheus A. Chaves, Samantha C. Pinho

**Affiliations:** ^1^ Laboratory of Encapsulation and Functional Foods (LEnAlis), Department of Food Engineering, School of Animal Science and Food Engineering (FZEA) University of São Paulo (USP) Pirassununga Brazil; ^2^ Laboratory of Molecular Morphophysiology and Development (LMMD), Department of Veterinary Medicine, School of Animal Science and Food Engineering (FZEA) University of São Paulo (USP) Pirassununga Brazil

## Abstract

Liposomes are lipid‐based delivery systems of interest in pharmaceutical, food, and nutraceutical industries due to their ability to encapsulate bioactive compounds, such as vitamins, improving stability, solubility, and bioavailability. This study aimed to develop functional yogurt enriched with vitamins D_3_ and B_12_ by encapsulating these micronutrients in liposomal dispersions produced with food‐grade phospholipids, coated with pectin, and subjected to lyophilization. An optimal sucrose concentration was selected as a cryoprotectant to minimize detrimental effects of freeze‐drying on vesicle integrity. Lyophilized vesicles were characterized by bioactive retention and physicochemical properties. High retention of both vitamins was observed across most formulations, except those containing only hydrogenated saturated phospholipids, which showed reduced encapsulation efficiency. These vesicles were incorporated into yogurt, and the enriched product was evaluated for bioactive content, physicochemical and rheological properties, and sensory acceptability. The addition of vesicles did not negatively affect yogurt stability or consumer acceptance. In vitro digestion using the INFOGEST 2.0 protocol revealed increased vitamin D_3_ bioaccessibility after the gastric phase only in samples containing Phospholipon 90G‐based liposomes; however, after the intestinal phase, all formulations showed enhanced availability. Vitamin B_12_ bioaccessibility remained consistent throughout digestion. Overall, results demonstrate a viable approach for coencapsulation and protection of hydrophobic and hydrophilic vitamins within a commonly consumed dairy product, offering a promising strategy for developing functional foods supporting daily nutritional supplementation.

**Practical Applications:**

Pectin‐coated and uncoated liposomes were successfully freeze‐dried with sucrose as a cryoprotectant. Purified phospholipids improved the bioaccessibility of vitamin D_3_ after gastric digestion. Liposome enrichment did not alter yogurt stability or sensory acceptance. The strategy allows the co‐administration of hydrophilic and hydrophobic vitamins in dairy products.

## 1 Introduction

The incorporation of bioactive compounds into food products is a well‐established strategy to enhance nutritional value and help prevent nutrient deficiencies. Among these compounds, vitamin D_3_ (VD3) and vitamin B_12_ (VB12) play vital roles in human metabolism, supporting bone health, immune regulation, and the maintenance of neurological function (Combs and McClung 2016). Despite their importance, the effective application of these vitamins remains challenging due to distinct physicochemical limitations. VD3 exhibits low aqueous solubility and poor chemical stability, whereas VB12, although water‐soluble, may still present stability and bioavailability constraints in complex food systems, collectively impairing their absorption upon oral delivery (Chaves et al. 2023).

To address these challenges, encapsulation technologies, particularly liposomal systems, have emerged as promising delivery platforms. Liposomes are spherical vesicles consisting of one or more phospholipid bilayers enclosing an aqueous core (Lasic and Papahadjopoulos 1998). This amphiphilic structure allows the simultaneous encapsulation of hydrophilic and hydrophobic compounds, enabling broad versatility for nutrient delivery (Emami et al. 2016; Chai and Park 2024). Their application in food has gained significant attention owing to their favorable properties, including low toxicity, high encapsulation efficiency, scalability, and ability to protect labile compounds from environmental degradation (Lavelli et al. 2021; Fan et al. 2023).

In recent years, the coencapsulation of multiple bioactive compounds within a single delivery system has been widely explored in the pharmaceutical sector for enhancing therapeutic efficacy through synergistic effects and targeted delivery. Translating this concept to food systems offers opportunities to simultaneously deliver essential nutrients, meeting growing consumer demand for functional foods with added health benefits (X. Liu et al. 2020; Shen et al. 2025). However, the success of coencapsulation depends largely on the production method employed. Among available techniques, ultrasonic homogenization has shown particular promise in food applications due to its capacity to produce uniformly sized vesicles in a solvent‐free, scalable, and efficient manner (Chotphruethipong et al. 2021; Pavlović et al. 2022; Sharma et al. 2023).

Despite their advantages, liposomes remain thermodynamically unstable and are susceptible to physicochemical degradation under environmental stresses (Frenzel and Steffen‐Heins 2015). To improve their structural integrity and functionality, surface modification with biopolymers has been investigated as a strategy to enhance liposome stability and regulate compound release during digestion. Various biopolymers, including sodium alginate (W. Liu et al. 2016), whey protein isolate (Frenzel et al. 2015), gelatin (Nunes et al. 2016), chitosan (Sebaaly et al. 2021), and pectin (Bhargavi et al. 2021; Ferreira et al. 2025), have been explored. Among these, pectin stands out due to its ability to resist gastric conditions while being selectively degraded in the intestinal tract (Bhargavi et al. 2021; Su et al. 2024). In parallel, lyophilization is widely used to enhance the shelf‐life and long‐term stability of liposomes. However, the lyophilization process can compromise vesicle integrity unless appropriate cryoprotectants, such as sucrose, are employed to preserve structural and functional properties (Toniazzo et al. 2017; Yu et al. 2021).

Yogurt, a widely consumed dairy product with proven nutritional benefits, serves as an ideal vehicle for delivering encapsulated bioactives. Nevertheless, successful fortification requires that the encapsulated nutrients remain bioaccessible upon ingestion. In vitro digestion models provide a controlled and standardized means to simulate gastrointestinal conditions and evaluate the release and potential absorption of nutrients from food matrices (Gonçalves et al. 2021; Faubel et al. 2023).

Overall, the incorporation of lyophilized, pectin‐coated liposomes coencapsulating VD3 and VB12 into yogurt represents a promising strategy for developing functional dairy products aimed at addressing both macro‐ and micronutrient deficiencies. This study aimed to coencapsulate these vitamins using ultra‐sonication, apply pectin coating, and subject the vesicles to lyophilization. The resulting dried liposomes were then incorporated into yogurt, and the impact of this incorporation on physicochemical properties during storage was assessed. In addition, in vitro digestion was conducted to evaluate the release and bioaccessibility of the encapsulated vitamins. The findings are expected to advance the development of fortified dairy products and provide insights into the feasibility and effectiveness of liposome‐based delivery systems in functional food applications.

## 2 Materials and Methods

### 2.1 Material

Liposomes were produced using two types of purified soy lecithin: Phospholipon 90G (P90G, >94% w/w phosphatidylcholine [PC], hydrogenated) and Lipoid S45 (PS45, >45% w/w PC, 10%–18% phosphatidyl‐ethanolamine [PE], nonhydrogenated), both of which were supplied by Lipoid GmbH (Ludwigshafen, Germany). Apple pectin (degree of esterification: 50%–75%), VD3 (cholecalciferol, >98% purity, analytical standard) and VB12 (cobalamin, ≥98% purity) were obtained from Sigma‐Aldrich (St. Louis, MO, USA). For in vitro digestion experiments, porcine pepsin (P7012), porcine pancreatin (P7545), bovine bile extract (B3883), hemoglobin from bovine blood (H2500), *p*‐toluene‐sulfonyl‐l‐arginine methyl ester (TAME, P4626), and Pefabloc SC (AEBSF) were used, all supplied from Sigma‐Aldrich. The ultrapure water used in all experiments was obtained from a Milli‐Q purification system (Millipore Corp., Billerica, MA, USA). All other chemicals used were of analytical reagent grade.

### 2.2 Methods

#### 2.2.1 Production of Liposomes Coencapsulating VD3 and VB12 and Pectin Coating

Liposomes were produced via ultrasonication according to Ferreira et al. (2025), with formulations detailed in Table 1. Phospholipid dispersions were obtained by dispersing either Phospholipon 90G or Lipoid S45 (5 mg/mL) in deionized water, followed by probe sonication using an ultrasonic tip homogenizer (SFX550, Branson, St. Louis, MO, USA) for 25 cycles (5‐s on, 2‐s off) at 60% amplitude. For coencapsulation, 1 mL of VD3 stock solution (2.5 mg/mL) was added to achieve 50 µg VD3/mL, and 4 mL of VB12 stock solution (0.25 mg/mL) was added for a final concentration of 20 µg VB12/mL.

**TABLE 1:** Composition of uncoated and pectin‐coated liposomal formulations containing vitamins D_3_ and B_12_.

	Components			
Formulation	Phospholipid (PL)	Pectin (mg/mg PL)	Vitamin D_3_ (µg/mg PL)	Vitamin B_12_ (µg/mg PL)
P90G	Phospholipon 90G	—	10	4
LS45	Lipoid S45	—	10	4
LS45‐R1	Lipoid S45	0.5	10	4
LS45‐R2	Lipoid S45	1.0	10	4

Pectin coating followed the method of Nguyen et al. (2011), with modifications by Ferreira et al. (2025). Pectin was dispersed in 5 mM phosphate buffer (pH 7.0) at concentrations of 2.5 and 5.0 mg/mL (R1 and R2, respectively) and stirred for 8 h at room temperature. Equal volumes (20 mL) of pectin and liposome dispersions were mixed under magnetic stirring, with pectin added at 1 mL/min using a peristaltic pump (Master Flex 7528‐30, Cole‐Parmer, Vernon Hills, IL, USA). Only LS45‐based liposomes were coated, as Ferreira et al. (2025) showed P90G‐based liposomes destabilized upon pectin addition. Despite this, P90G formulations retained high bioactive content and were thus included for comparison in food matrix incorporation and in vitro digestion.

#### 2.2.2 Lyophilization of Liposomes

Liposome dispersions were frozen in liquid nitrogen (−210°C for 2 min) and freeze‐dried in a workbench lyophilizer (L101‐Liotop, São Carlos, Brazil) for 72 h at −53°C under 444 µHg vacuum. Shelf temperature was not actively controlled in this system; therefore, primary and secondary drying occurred continuously under constant operating conditions until mass stabilization. Sucrose was added as a cryoprotectant at phospholipid‐to‐sucrose ratios of 1:2, 1:3, and 1:4 (w/w), based on established protocols to preserve bilayer integrity during lyophilization (Toniazzo et al. 2017; Yoshida et al. 2010). Dried samples were stored in a desiccator with silica gel at 25°C for up to 4 weeks.

#### 2.2.3 Reconstitution of Lyophilized Liposomes

Lyophilized powders were reconstituted in deionized water (pH 6.8 ± 0.1; negligible ionic strength) to their original mass concentration using a mechanical stirrer with a three‐blade naval propeller at 100 rpm and room temperature. Samples were collected at 10, 20, and 30 min to assess hydrodynamic diameter and size distribution, determining rehydration efficiency and optimal reconstitution time. After full rehydration, particle size, distribution, and zeta potential were analyzed.

##### 2.2.3.1 Hydrodynamic Mean Diameter, Size Distribution, And Zeta Potential

Hydrodynamic mean diameter and particle size distribution were measured via photon correlation spectroscopy (PCS) using a ZetaSizer Nano ZS90 (Malvern Instruments, UK). To minimize multiple light scattering, samples were diluted 15‐fold in deionized water. Measurements were performed at 25°C using a 627 nm He–Ne laser at a 90° detection angle. Zeta potential was assessed using a ZetaPlus analyzer based on electrophoretic mobility. All analyses were performed in triplicate, with each value representing the average of 10 readings.

#### 2.2.4 Characterization of Lyophilized Liposomes Coencapsulating VD3 and VB12

##### 2.2.4.1 Moisture Content, Water Activity, and Hygroscopicity

Moisture content was determined using an infrared moisture analyzer (MB35, Ohaus, USA) at 105°C with 0.5 g of sample. Water activity (*A*_w_) was measured at 25°C using a dew point water activity meter (AquaLab 3TE, Decagon Devices, USA). Hygroscopicity was evaluated following Cai and Corke (2000), with modifications. Briefly, 0.2 g of sample was stored in desiccators (RH = 81.1%) for 1 week. Results were then expressed as g water adsorbed/100 g dry matter. Equilibrium moisture content (*H*_eq_) was determined gravimetrically and calculated using Equation (1), where *m*_water_ is the sum of initial moisture and adsorbed water, and *m*_dry_ is dry mass.

(1) $$H_{\mathrm{eq}}=m_{\mathrm{water}}/m_{\mathrm{dry}}$$

##### 2.2.4.2 Instrumental Colorimetry

The color of lyophilized liposomes was measured using a noncontact colorimeter (AEROS, HunterLab, VA, USA) in the CIEL*a*b* color space, following the *Commission Internationale de l'Eclairage* (CIE) guidelines, with a D65 illuminant and 10° observation angle. Measured parameters included lightness (L*) and chromatic coordinates (a*, b*). Chroma (C*_ab_) and hue angle (h*_ab_) were calculated using Equations (2) and (3), respectively. The total color difference (∆*E*) between Days 1 and 120 of storage was calculated via Equation (4), using initial *L*_0_*, *a*_0_*, and *b*_0_* values.

(2) $$C_{\mathrm{ab}}^{*}=\sqrt{{\left(a^{*}\right)}^{2}+{\left(b^{*}\right)}^{2}}$$

(3) $$h_{\mathrm{ab}}^{*}=\mathrm{ta}n^{-1}\;\left(b^{*}/a^{*}\right)\;$$

(4) $$\Delta E=\sqrt{{\left(L^{*}-\;L_{0}^{*}\right)}^{2}+{\left(a^{*}-\;a_{0}^{*}\right)}^{2}+{\left(b^{*}-\;b_{0}^{*}\right)}^{2}}$$

##### 2.2.4.3 Quantification of VD3 and VB12

###### 2.2.4.3.1 Methanolic Extract Preparation

To quantify encapsulated vitamins, 0.3 g of lyophilized sample was mixed with 5 mL methanol, vortexed for 5 min (Multi Reax Heidolph, Germany), and sonicated for 10 min (Model 410US, Marte, Brazil). Samples were centrifuged at 7000 × *g* for 5 min at 4°C (5430R, Eppendorf, Germany). The supernatant was collected, and extraction was repeated to maximize recovery. Combined extracts were filtered (0.45 µm nylon membrane, 13 mm i.d.) into 2 mL amber vials and stored for high‐performance liquid chromatography (HPLC) analysis. Aliquots of the same methanolic extract were subsequently used for the independent HPLC quantification of VD3 and VB12 under their respective chromatographic conditions.

###### 2.2.4.3.2 VD3 Quantification via HPLC

VD3 was quantified following the method by Staffas et al. (2003), with adaptations. A Shimadzu Prominence HPLC system (Kyoto, Japan) with LC Solution software (v1.25) was used, equipped with quaternary pump, degasser, auto‐injector, column oven, and diode array detector. Separation was performed on a Shim‐Pack VP‐ODS column (250 × 4.6 mm, 4.6 µm) at 35°C. The mobile phase (methanol:acetonitrile, 9:1 v/v) ran at 0.60 mL/min. A 30 µL sample was injected, and detection occurred at 265 nm. Runtime was 10 min. VD3 identification was based on retention time (7.30 min), and quantification used external calibration with seven standards (4.5–36 µg/mL), injected in triplicate. This method is routinely employed by our group for VD3 in liposomal systems (Chaves et al. 2018; Chaves and Pinho 2020).

###### 2.2.4.3.3 VB12 Quantification via HPLC

VB12 was quantified using a modified version of the method by Bochicchio et al. (2016), employing the same Shimadzu Prominence HPLC system controlled by LC Solution software (v1.25). Separation was carried out on a Kinetex EVO C18 column (150 × 4.6 mm, 5.0 µm; Phenomenex, USA) at 35°C. The mobile phase, water:acetonitrile (87.5:12.5 v/v) with 0.02% formic acid, flowed at 0.60 mL/min. Each sample (10 µL) was injected and detected at 362 nm over a 10 min runtime. Identification was based on retention time (5.14 min) compared with a VB12 standard. Quantification used external calibration from peak areas of six standard solutions (4.3–21.2 µg/mL), injected in duplicate.

##### 2.2.4.4 Scanning Electron Microscopy

Morphology of lyophilized liposomes was assessed using a HITACHI TM‐300 Tabletop SEM (Tokyo, Japan). Samples were mounted on carbon adhesive tape on aluminum stubs without coating. Images were taken at 15 kV and 200× magnification.

#### 2.2.5 Yogurt Production With Vitamin‐Loaded Lyophilized Liposomes

Yogurts enriched with VD3‐ and VB12‐loaded lyophilized liposomes were produced by adding 2% (w/w) liposomal powder to commercial whole yogurt (Nestlé S.A.), followed by manual stirring (∼5 min) until homogenized. Post‐fermentation incorporation was selected to preserve liposomal integrity and vitamin stability and to reflect a feasible industrial fortification strategy for fermented dairy products. Five formulations were evaluated: (i) blank (unfortified yogurt); (ii) Y90G (containing P90G liposomes); (iii) YS45 (containing LS45 liposomes); (iv) YR1 (containing LS45 liposomes with 2.5 mg/mL pectin); and (v) YR2 (containing LS45 liposomes with 5.0 mg/mL pectin). Each formulation, including the unfortified control (blank), was produced in triplicate, transferred to identical glass containers, hermetically sealed, and stored at 10°C until further analysis.

#### 2.2.6 Characterization of Yogurt Containing Vitamin‐Loaded Lyophilized Liposomes

##### 2.2.6.1 Physicochemical Characterization

Yogurt pH was measured using a pH meter (Digimed, Brazil). Water activity was determined as per Section 2.2.4.1. Titratable acidity was determined by titrating 10 g of yogurt diluted in 10 mL of deionized water with 0.1 N NaOH to pH 8.2 (AOAC 1990). Results were calculated using Equation (5) and expressed as g lactic acid/100 g yogurt.

(5) $$\%\;\mathrm{titratable}\;\mathrm{acidity}=\left(V_{\mathrm{NaOH}}\times f\times 0.9\right)/P$$

where *V*_NaOH_ is the titrant volume (mL), *f* is the correction factor, 0.9 is the lactic acid conversion factor, and *P* is sample mass (g).

##### 2.2.6.2 Degree of Syneresis (%*S*)

Syneresis was determined by placing 30 g of yogurt on filter paper in a funnel over an Erlenmeyer flask, refrigerated at 8°C for 5 h. The expelled whey was weighed, and syneresis (%) calculated as shown in Equation (6).

(6) $$\%S=\left[\mathrm{mass}\;\mathrm{ofexpelled}\;\mathrm{whey}\;\left(g\right)/\mathrm{mass}\;\mathrm{of}\;\mathrm{yogurt}\;\left(g\right)\right]\times 100$$

##### 2.2.6.3 Instrumental Colorimetry

Color changes, influenced by VB12, were evaluated during storage using instrumental colorimetry. Samples were placed in Petri dishes, and color parameters were measured under the same conditions described for the lyophilized liposomes (Section 2.2.4.2).

##### 2.2.6.4 Proximate Composition

Proximate composition included protein (AOAC 1990), lipid (Bligh and Dyer 1959), moisture, and ash contents (Instituto Adolfo Lutz [IAL] 2008). Carbohydrates were calculated by difference from the total mass.

##### 2.2.6.5 Rheological Characterization

Rheological behavior was assessed as per Sah et al. (2016), using a rotational rheometer (AR2000, TA Instruments, USA) with cone‐plate geometry (2°, 60 mm). Measurements were performed at 10°C followed by 2 min of equilibration. Shear rate (0.01–100 s^−1^) was applied under steady‐state conditions, and flow curves were fitted to the Herschel–Bulkley model, described by Equation (7):

(7) $$\tau =\tau_{0}+\gamma \times K^{n}$$

where *τ* is shear stress (Pa), *τ*_0_ is yield stress (Pa), *K* is the consistency coefficient (Pa s*^n^*), and *n* is the flow behavior index (dimensionless).

Oscillatory tests assessed viscoelasticity. The linear viscoelastic region (LVR) was first identified at 10°C (strain: 0.01% to 15%, 1 Hz). Storage (*G*′) and loss (*G*″) moduli were then measured. Frequency sweeps (0.1–10 Hz) were performed at 0.1% strain to characterize frequency‐dependent viscoelastic behavior.

##### 2.2.6.6 Determination of VD3 and VB12 in yogurt

To quantify VD3 and VB12, 8 g of yogurt was homogenized with 9 mL methanol using a high‐speed homogenizer (T25, IKA, Germany) at 9000 rpm for 1 min, followed by 10 min vortexing and 10 min ultrasonic treatment. After centrifugation (7100 × *g*, 5 min, 4°C), the supernatant was collected. The residue was re‐extracted using the same procedure, and the supernatants were pooled, vortexed, sonicated, and treated with 50 µL formic acid for protein precipitation. After a final centrifugation, the extract was filtered (0.45 µm) and transferred to chromatographic vials. Vitamin quantification was performed via HPLC as described in Sections 2.2.4.3.2 and 2.2.4.3.3.

##### 2.2.6.7 Microbiological Characterization

Microbiological analyses included counts of lactic acid bacteria, yeasts, molds, coliforms and *Escherichia coli* using APHA methods (Reasoner 2004). Five serial dilutions per sample were prepared in 0.1% peptone water. Lactic acid bacteria were plated (10^−4^–10^−6^) on MRS agar using the pour plate technique and incubated at 37 ± 1°C for 48 ± 3 h. Results were expressed as CFU/g. Yeast and molds were plated (10^−1^–10^−3^) on DRBC agar and incubated at 25 ± 1°C for 5 days. Coliforms and *E. coli* were assessed with Compact Dry EC plates.

##### 2.2.6.8 Static In Vitro Digestion, Vitamin Release, and Protein Hydrolysis

In vitro digestion followed the standardized INFOGEST 2.0 protocol (Brodkorb et al. 2019), with preliminary verification of enzyme activity and bile salt concentration. Digestion proceeded through three phases:

- Oral phase (OP): 5 g yogurt was mixed with 4.975 mL simulated salivary fluid (SSF) and 25 µL of CaCl_2_·2H_2_O (0.3 M), stirred at 37°C for 2 min.
- Gastric phase (GP): 8 mL of simulated gastric fluid (SGF), 0.5 mL of pepsin (2000 U/mL), 5 µL of CaCl_2_·2H_2_O (0.3 M), and deionized water (to 20 mL) were added to the oral bolus. The pH was adjusted to 3.0, and the mixture incubated at 37°C for 2 h at 200 rpm.
- Intestinal phase (IP): 8.5 mL of simulated intestinal fluid (SIF), 5 mL of pancreatin (trypsin activity: 100 U/mL), 2.5 mL of bile extract (10 µM), 40 µL of CaCl_2_·2H_2_O (0.3 M), and deionized water (to 40 mL) were added to the gastric chyme. pH was adjusted to 7.0, and incubation continued at 37°C for 3 h at 200 rpm.

Post‐digestion, samples were flash‐frozen in liquid nitrogen. VD3 and VB12 concentrations were analyzed via HPLC (Sections 2.2.4.3.2 and 2.2.4.3.3). Protein hydrolysis was quantified using the OPA method (Nielsen et al. 2001) with absorbance measure at 240 nm using a microplate spectrophotometer (Benchmark Plus; Bio‐Rad, Hercules, USA).

##### 2.2.6.9 Sensorial Evaluation

The sensory evaluation protocol was approved by the Research Ethics Committee of FZEA/USP (CAAE: 69942223.9.0000.5422). A total of 123 untrained consumers, all of whom signed informed consent, participated in the yogurt evaluation. Two methods were used: an affective test and a check‐all‐that‐apply (CATA) approach. In the affective test, participants rated appearance, color, texture, flavor, aroma, and overall acceptability on a 9‐point hedonic scale (1 = *dislike extremely* to 9 = *like extremely*) and indicated purchase intent. In the CATA method, adapted from Cadena et al. (2014) and Brito‐Oliveira et al. (2024), participants selected terms from a predefined list describing yogurt attributes: sweet, soft, fluid, sticky, lumpy, homogeneous, heterogeneous, viscous, watery, sour, aftertaste, creamy, liquid, consistent, and pinkish. CATA data were expressed as the percentage of consumers who selected each attribute for each sample. The frequencies were statistically analyzed using Cochran's *Q* test (*p* < 0.05) to determine overall differences among samples for each descriptor. When significant differences were detected, pairwise comparisons were performed using McNemar's test (*p* < 0.05) to identify which samples differed from each other. All samples were microbiologically tested (total coliforms, *E. coli*, yeasts, and molds) to ensure safety before evaluation.

#### 2.2.7 Statistical Analysis

All measurements were performed in triplicate, and results are reported as mean ± standard errors. Data were analyzed using ANOVA, followed by Tukey's test at a 5% significance level, in SAS software v 9.2 (SAS Institute Inc., NC).

## 3 Results and Discussion

### 3.1 Effect of Cryoprotectant Addition on Liposome Lyophilization

This section evaluates the physicochemical stability of liposomes made from different phospholipids after lyophilization and rehydration, with a focus on their suitability for yogurt fortification. Stability upon rehydration is essential to ensure proper dispersion, homogeneity, and functional integrity within food matrices.

Table 2 summarizes the physicochemical parameters obtained for liposome dispersions after lyophilized rehydration. Without cryoprotectant, lyophilized P90G liposomes showed significant size increase (>2000 nm) and high PDI (>0.5), indicating aggregation and heterogeneity due to bilayer disruption during freeze‐drying (Susa et al. 2021). The addition of sucrose (2:1 w/w) reduced particle size and stabilized PDI (∼0.3), suggesting effective protection. Higher sucrose concentrations did not yield further improvements. Rehydration time (10–30 min) had no significant impact, suggesting rapid and consistent reconstitution behavior across time points. The size reduction may result from sucrose forming a vitrified matrix that prevents vesicle fusion and stabilizes the bilayer (Deng et al. 2023). Zeta potential decreased post‐rehydration in all samples, possibly due to surface charge changes after freeze‐drying or sucrose adsorption (Elhissi et al. 2010).

**TABLE 2:** Physicochemical parameters obtained for the liposome dispersions before and after lyophilized rehydration.

Liposome composition	Before lyophilization			Sugar‐to‐lipid mass ratio	Stirring time for reconstitution								
					10 min			20 min			30 min		
	Average diameter (nm)	PDI	ZP		Average diameter (nm)	PDI	ZP	Average diameter (nm)	PDI	ZP	Average diameter (nm)	PDI	ZP
P90G	257.0^B^ ± 6.0	0.28^C^ ± 0.01	−10.0^A^ ± 1.0	—	—	—	—	—	—	—	—	—	—
				—	2589^A,a^ ± 271	0.46^B,a^ ± 0.09	−39.4^D,b^ ± 4.0	2480^A,a^ ± 433	0.52^AB, a^ ± 0.06	−39.0^D, b^ ± 1.8	2837^A,a^ ± 426	0.6^A,a^ ± 0.14	−38.0^D, c^ ± 1.2
				2:1^*^	188^B,b^ ± 3.3	0.32^C,b^ ± 0.03	−18.0^B,a^ ± 4.4	188^B,b^ ± 2.8	0.33^C,b^ ± 0.03	−16.7^B,a^ ± 4.8	188^B,b^ ± 2.9	0.31^C,b^ ± 0.02	−21.4^BC,a^ ± 2.0
				3:1	188^B,b^ ± 3.0	0.31^C,b^ ± 0.01	−22.7^BC,a^ ± 1.5	186^B,b^ ± 2.0	0.33^C,b^ ± 0.03	−21.0^BC,a^ ± 1.2	188^B,b^ ± 2.0	0.31^C,b^ ± 0.03	−19.9^BC,a^ ± 0.8
				4:1	180^B,b^ ± 5.6	0.30^C,b^ ± 0.03	−22.4^BC,a^ ± 3.5	178^B,b^ ± 3.4	0.28^C,b^ ± 0.03	−22.3^BC,a^ ± 5.3	180^B,b^ ± 3.7	0.30^C,b^ ± 0.02	−24.8^C,b^ ± 1.7
LS45	132^C^ ± 8.4	0.23^B^ ± 0.02	−63.0^ABCD^ ± 7.1	—	—	—	—	—	—	—	—	—	—
				—	n.r.	n.r.	n.r.	n.r.	n.r.	n.r.	989^A,a^ ± 54	0.51^A,a^ ± 0.04	−55.5^A,a^ ± 1.4
				2:1^*^	102^C,b^ ± 1.8	0.24^B,a^ ± 0.01	−62.6^ABC,a^ ± 1.8	101^C,b^ ± 0.6	0.23^B,b^ ± 0.02	−64.2^ABCD,a^ ± 3.1	102^C,c^ ± 1.5	0.24^B,b^ ± 0.01	−63.7^ABCD,a^ ± 3.1
				3:1	146^C,b^ ± 7.6	0.52^A,b^ ± 0;09	−67.1^BCD,a^ ± 3.1	144^C,b^ ± 2.8	0.54^A,a^ ± 0.11	−70.1^CD,a^ ± 6.8	147^C,c^ ± 3.0	0.54^A,a^ ± 0.13	−73.0^D,b^ ± 8.4
				4:1	644^B,a^ ± 169	0.74^A,a^ ± 0.19	−61.4^ABC,a^ ± 5.8	623^B,a^ ± 138	0.64^A,a^ ± 0.17	−62.3^ABC,a^ ± 4.5	653^B,b^ ± 162	0.70^A,a^ ± 0.18	−59.4^AB,a^ ± 2.2
Pectin‐coated formulations													
LS45‐R1	130^C^ ± 1.2	0.34^B^ ± 0.03	−24.7^A^ ± 2.3	—	—	—	—	—	—	—	—	—	—
				—	776^AB,a^ ± 53	0.55^AB,b^ ± 0.09	−60.6^C,c^ ± 2.6	817^A,a^ ± 15	0.56^AB,b^ ± 0.02	−61.^8CD,b^ ± 1.6	824^A,a^ ± 40	0.58^A,a^ ± 0.07	−62.3^CD,b^ ± 1.9
				2:1^*^	656A^B,b^ ± 104	0.75^A,a^ ± 0.12	−52.7^B,a^ ± 1.2	673^AB,a^ ± 148	0.68^A,ab^ ± 0.16	−54.8^B,a^ ± 0.8	625^B,b^ ± 158	0.74^A,a^ ± 0.17	−54.1^B,a^ ± 4.1
				3:1	657^AB,b^ ± 40	0.66^A,ab^ ± 0.12	−54.9^B,a^ ± 2.3	698^AB,a^ ± 67	0.74^A,a^ ± 0.10	−54.9^B,a^ ± 1.7	727^AB,ab^ ± 117	0.77^A,a^ ± 0.13	−54.4^B,a^ ± 1.3
				4:1	793^AB,a^ ± 29	0.56^AB,b^ ± 0.09	−65.6^D,d^ ± 2.2	816^A,a^ ± 45	0.58^A,ab^ ± 0.04	−62.4^CD,b^ ± 2.6	774^AB,ab^ ± 76	0.56^AB,a^ ± 0.13	−64.7^CD,b^ ± 1.4
LS45‐R2	146^E^ ± 1.5	0.39^D^ ± 0.01	−25.7^A^ ± 1.4	—	—	—	—	—	—	—	—	—	—
				—	543^D,b^ ± 8.2	0.56^ABC,ab^ ± 0.09	−61.1^FG,c^ ± 2.8	568^CD,a^ ± 19	0.54^ABCD,a^ ± 0.02	−62.1^G,b^ ± 1.6	597^BCD,a^ ± 45	0.52^ABCD,ab^ ± 0.03	−59.5^EFG,b^ ± 2.5
				2:1^*^	641^ABCD,ab^ ± 104	0.48^BCD,b^ ± 0.03	−58.0^DEFG,bc^ ± 3.4	639^ABCD,a^ ± 126	0.59^ABC,a^ ± 0.08	−52.3^BCD,a^ ± 4.0	620^ABCD,a^ ± 99	0.53^ABCD,ab^ ± 0.07	−52.7^BCDE,a^ ± 2.8
				3:1	756^A,a^ ± 78	0.67^A,a^ ± 0.13	−53.2^BCDE,ab^ ± 4.0	686^ABCD,a^ ± 24	0.60^ABC,a^ ± 0.09	−54.9^CDEF,a^ ± 1.0	683^ABCD,a^ ± 84	0.64^AB,a^ ± 0.11	−52.8^BCDE,a^ ± 2.2
				4:1	736^AB,a^ ± 31	0.50^BCD,b^ ± 0.06	−47.7^B,a^ ± 4.9	679^ABCD,a^ ± 73	0.53^ABCD,a^ ± 0.07	−52.5^BCD,a^ ± 1.4	709^ABC,a^ ± 39	0.48^CD,b^ ± 0.04	−49.4^BC,a^ ± 5.2

*Note*: Averages followed by the same capital letter were not significantly different (*p* > 0.05) for samples with the same liposome composition. Averages followed by the same lowercase letter in the same column were not significantly different (*p* > 0.05) according to Tukey's test.

Abbreviations: n.r., not recorded (rehydration was not observed under the tested conditions); PDI, polydispersity index; ZP, zeta potential.

*Sugar‐to‐lipid ratio selected for continuation in subsequent stages of the study.

Uncoated LS45 liposomes were also unstable without cryoprotectant, requiring at least 30 min of re‐hydration to form dispersions, which remained large (989 nm) and heterogeneous (PDI = 0.51). Sucrose addition (2:1 w/w) improved stability and reduced size; however, higher concentrations (3:1 and 4:1 w/w) increased PDI and size, indicating excessive sucrose may hinder rehydration. As with P90G, rehydration time had minimal influence when sucrose was present. LS45 formulations had more negative zeta potentials than P90G, likely due to higher phospholipid unsaturation, which increases surface exposure of negatively charged phosphate groups (Chun et al. 2013).

Pectin‐coated liposomes showed marked size increases after lyophilization and rehydration, regardless of sucrose concentration. The highest sucrose level (4:1 w/w) failed to prevent size enlargements, suggesting pectin may interfere with cryoprotective effects due to steric hindrance or interactions during freezing (Ma et al. 2025). PDIs were >0.3 before and >0.5 after lyophilization, reflecting pronounced size heterogeneity. Zeta potential consistently decreased post‐rehydration, regardless of cryoprotectant or coating. No significant differences (*p* < 0.05) were observed between coating levels (R1 vs. R2) or rehydration durations, indicating pectin concentration had minimal impact on stability once cryoprotectant was present.

**FIGURE 1:**
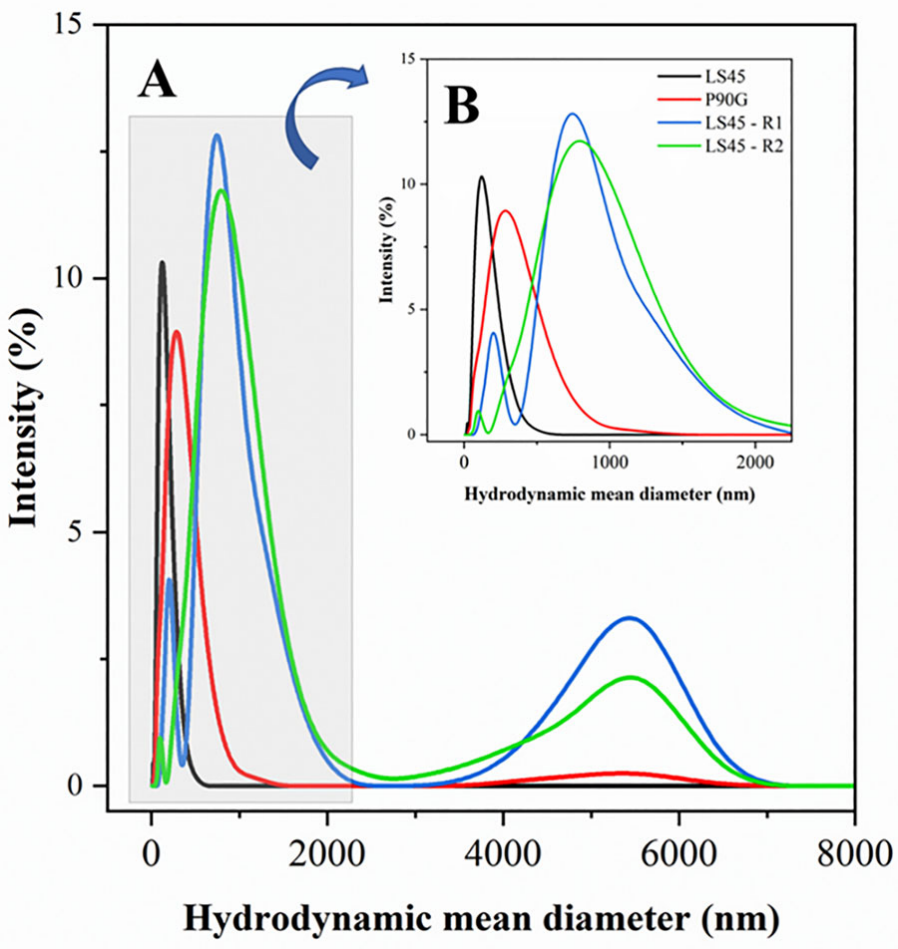
Size distribution profiles of liposomes formulated with Phospholipon 90G (P90G), Lipoid S45 (LS45), and Lipoid S45 coated with pectin (designated LS45‐R1 and LS45‐R2) reconstituted by magnetic stirring for 30 min. A sugar‐to‐lipid mass ratio of 2:1 was used in all formulations. R1 and R2 correspond to the addition of 2.5 and 5.0 mg/mL pectin, respectively, during liposome production. B provides a magnified view of the initial segment of the size distribution curves presented in A.

Therefore, sucrose at a 2:1 w/w ratio and 30 min rehydration were established as optimal, maintaining or improving the physicochemical stability of both coated and uncoated liposomes. Size distribution data (Figure 1) confirmed monomodal, narrow peaks for LS45, and broader yet acceptable profiles for P90G. Pectin‐coated liposomes displayed multimodal distributions with peaks in the 4–6 µm range, but these sized remain below oral detection thresholds and are unlikely to affect yogurt texture or mouthfeel (Santagiuliana et al. 2019).

### 3.2 Production and Characterization of Lyophilized Liposomes Coencapsulating VD3 and VB12

#### 3.2.1 Visual Appearance and Morphology

**FIGURE 2:**
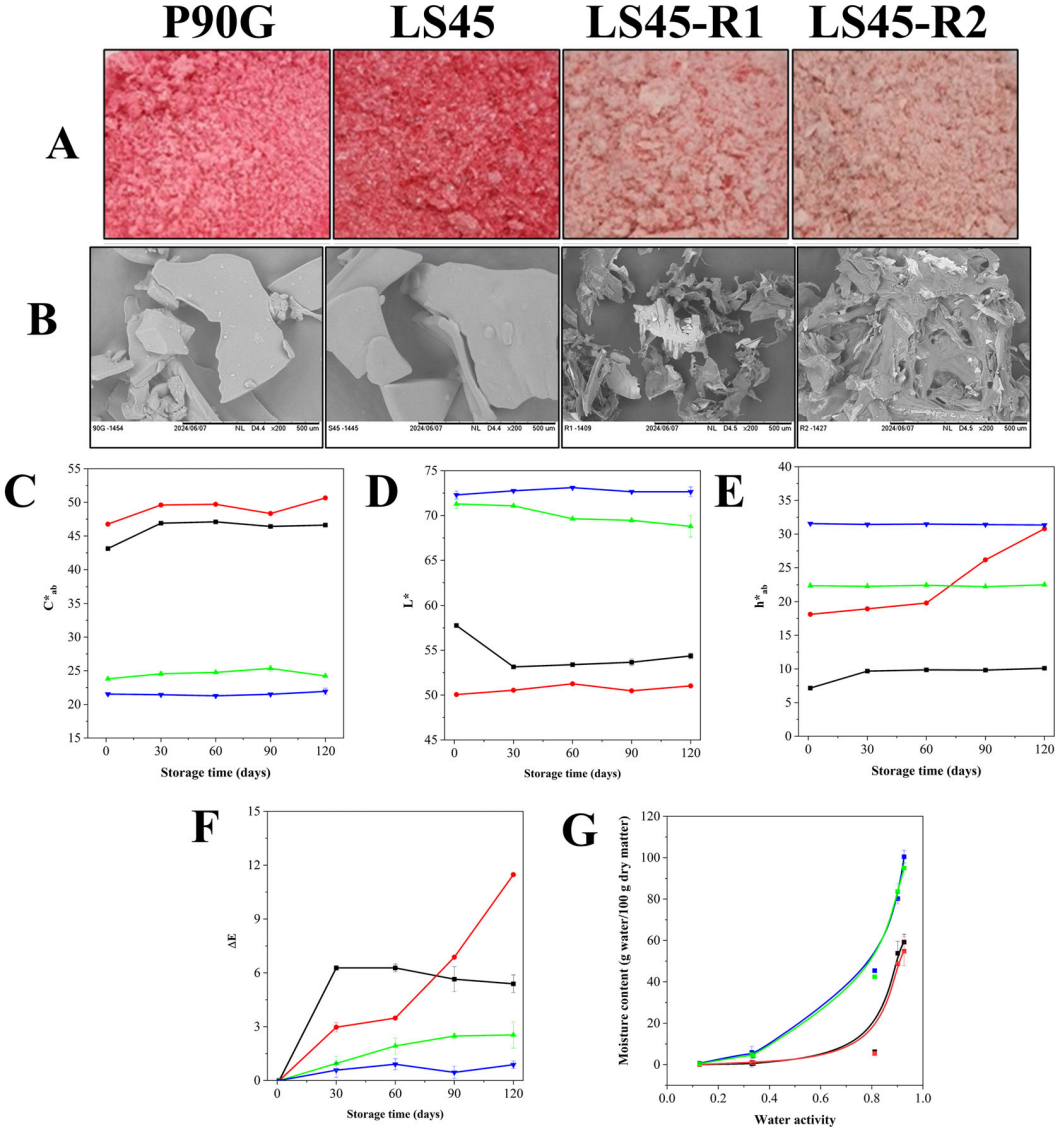
(A) Visual appearance; (B) scanning electron micrographs (SEM), colorimetric parameters: (C) chroma—C*_ab_, (D) hue angle—h*_ab_, and (E) total color difference—Δ*E*, and (F) water sorption isotherms fitted by the Oswin model for lyophilized liposomes coencapsulating vitamins D_3_ and B_12_. Liposomes were produced via Phospholipon 90G (P90G, red line), Lipoid S45 (LS45, black line), LS45‐R1 (blue line), and LS45‐R2 (green line). R1 and R2 correspond to the addition of 2.5 and 5.0 mg/mL pectin, respectively, during liposome production.

Figure 2A shows lyophilized liposomes coloaded with VD3 and VB12, all appearing pink due to VB12. P90G formulation had a lighter, more vivid pink than those with LS45. Both uncoated types appeared crystalline, likely due to sucrose used as a cryoprotectant. In contrast, pectin‐coated samples had a paler pink hue and a cotton‐like texture, likely because the pectin matrix inhibited crystal formation, as no visible sucrose crystals were observed. Scanning electron microscopy (SEM) images (Figure 2B) showed that P90G and LS45 produced amorphous, flake‐like granules, while LS45‐R1 and LS45‐R2 formed clusters of sharp, interconnected granules. This structure aligns with findings that pectin with ionized carboxyl groups cross‐links during drying, forming a dense fibrous matrix (Chen et al. 2022). A higher pectin content in LS45‐R2 led to more amorphous structures and fewer crystalline areas, consistent with behavior reported for lyophilized pectin‐/sugar‐rich powders (Conceição et al. 2016; Stachowiak et al. 2021).

#### 3.2.2 Colorimetric Stability

Colorimetric analysis confirmed formulation differences. Parameters C*_ab_, h*_ab_, and Δ*E* (calculated from a* and b* in Table S1) tracked color changes during storage. Chroma (C*_ab_, Figure 2C), indicating color vividness, increased in uncoated samples, especially in LS45 (46.76–50.66 by Day 120), suggesting reddish‐orange pigment transformation. P90G also increased slightly (43.13–46.63), though remained lower than LS45. Coated samples showed much more stable C*_ab_ values: LS45‐R1 peaked at 25.36 on Day 90, while LS45‐R2 remained nearly constant (21.54–21.93), suggesting pectin limited the exposure of VB12 to degradative factors. Hue angle (h*_ab_, Figure 2D), indicating color tone, remained low for P90G (7.16–10.11), reflecting a stable red hue. LS45 shifted from 18.12 to 30.80, moving toward orange–yellow, consistent with the C*_ab_ increase and pigment transformation. Coated samples stayed stable: LS45‐R1 around 22° and LS45‐R2 near 31.5°, showing the coatings' protective effect against tone changes. Total color difference (Δ*E*, Figure 2E) quantifies visual change. Values above 3 are noticeable (Fontes et al. 2014). LS45 had the largest change (Δ*E* from 0 to 11.48), indicating significant degradation. P90G reached Δ*E* ≈ 5–6 by Day 30. Coated samples had lower Δ*E* values: LS45‐R1 rose from 0.95 to 2.54, remaining under the visibility threshold until the end. LS45‐R2 stayed below 1, denoting imperceptible changes. Altogether, results show that pectin coatings significantly reduce color shifts and help maintain visual integrity during storage.

#### 3.2.3 Water Activity, Moisture Content, and Hygroscopicity

Table 3 shows the results for *A*_w_ and moisture content. Compared to P90G (0.28 ± 0.04) and LS45 (0.26 ± 0.04), LS45‐R1 and LS45‐R2 had significantly lower *A*_w_ values (0.14 ± 0.02), suggesting enhanced barrier properties from pectin coating. All samples stayed below the 0.6 *A*_w_ threshold, indicating water was bound within the matrix (Damodaran 2017). Moisture content varied from 3.30% to 4.74%, with no significant differences (*p* > 0.05), and the slightly higher values in LS45‐R1 and R2 did not raise *A*_w_, reinforcing that *A*_w_ and moisture content are not directly correlated. Hygroscopicity, however, was notably higher in pectin‐coated samples (44.6 and 41.3 g H_2_O/100 g dry matter) compared to uncoated ones, likely due to increased hydrophilic content. This is typical in polysaccharide‐rich powders, which should be stored under low‐*A*_w_ conditions to limit moisture uptake.

**TABLE 3:** Physicochemical parameters of lyophilized liposomes formulated with Phospholipon 90G (P90G), Lipoid S45 (LS45), and Lipoid S45 coated with pectin (designated LS45‐R1 and LS45‐R2). R1 and R2 correspond to the addition of 2.5 and 5.0 mg/mL pectin, respectively, during liposome production.

	Formulations			
Parameters	P90G	LS45	LS45‐R1	LS45‐R2
Water activity (*A*_w_)	0.28^A^ ± 0.04	0.26^A^ ± 0.04	0.14^B^ ± 0.02	0.14^B^ ± 0.02
Moisture content (%)	3.30^A^ ± 0.39	3.39^A^ ± 0.42	4.43^A^ ± 1.11	4.74^A^ ± 0.47
Hygroscopicity (g absorbed water/100 g d.m.)	4.08^C^ ± 0.45	2.94^D^ ± 0.06	44.6^A^ ± 0.69	41.3^B^ ± 0.15
Vitamin concentration—fresh liposomes (µg VD3/mg lyophilized liposomes)	2.6 ± 0.5	2.5 ± 0.2	1.9 ± 0.0	1.7 ± 0.0
Vitamin D_3_ retention—4 months of storage (%)	2	100	100	100
Vitamin concentration—fresh liposomes (µg VB12/mg lyophilized liposomes)	0.8 ± 0.1	1.0 ± 0.1	0.6 ± 0.0	0.6 ± 0.0
Vitamin B_12_ retention—4 months of storage (%)	28	81	100	100

*Note*: Averages followed by the same capital letter in the same column were not significantly different (*p* > 0.05) according to Tukey's test.

#### 3.2.4 Retention of Vitamins During Storage

**FIGURE 3:**
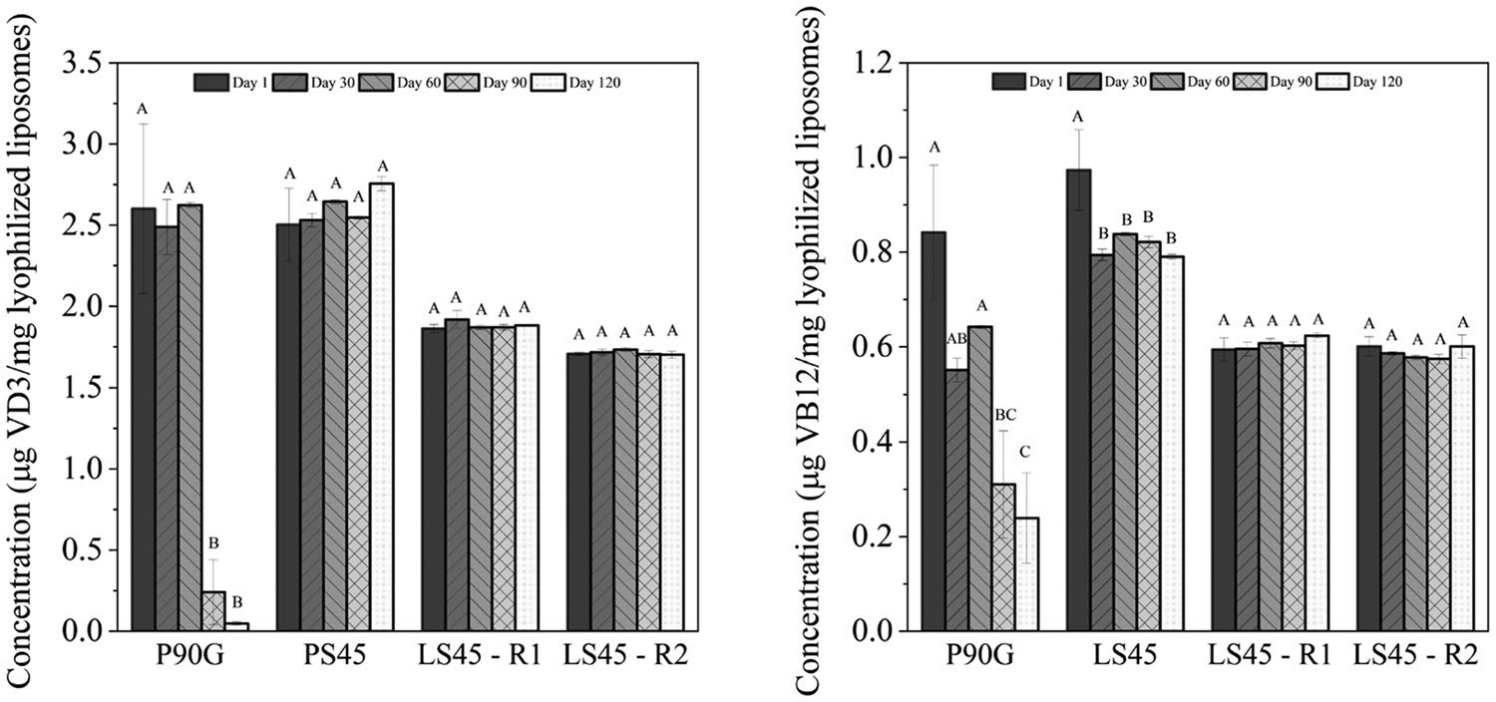
Temporal profiles of vitamin D_3_ and B_12_ concentrations in lyophilized liposomes formulated with Phospholipon 90G (P90G), Lipoid S45 (LS45), and Lipoid S45 coated with pectin (designated LS45‐R1 and LS45‐R2). R1 and R2 correspond to the addition of 2.5 and 5.0 mg/mL pectin, respectively, during liposome production. Means followed by the same capital letter represent nonsignificant differences (*p* < 0.05) between different formulations on the same day of storage. Means followed by the same lowercase letter represent nonsignificant differences (*p* < 0.05) between the same formulations over the storage time.

The stability of VD3 and VB12 in lyophilized liposomes was evaluated over 120 days (Figure 3; Table 3). VD3 retention varied notably by formulation. P90G showed rapid degradation, retaining only 2% by Day 120. In contrast, LS45‐based formulations, both uncoated and pectin‐coated, maintained 100% of their VD3, despite slightly lower initial concentrations likely due to dilution during coating and drying. VB12 followed a similar trend: P90G maintained just 28%, while uncoated LS45 remained 81%, with losses occurring mainly early in storage. Pectin‐coated formulations preserved 100% of VB12, highlighting the strong protective effect of the pectin matrix. This improved retention is likely due to the film‐forming and barrier proper‐ties of high‐methoxylated pectin, which limits vitamin exposure to degradation factors (Lopes et al. 2017; Hamadou et al. 2022; Su et al. 2024). Despite initial dilution, pectin coating significantly enhanced long‐term vitamin stability.

### 3.3 Characterization of Yogurt Containing Lyophilized Liposomes Coencapsulating VD3 and VB12

#### 3.3.1 Proximate Composition

Incorporating lyophilized liposomes coencapsulating VD3 and VB12 into yogurt altered its proximate composition as summarized in Table 4. Despite these changes, all formulations complied with Brazilian regulations on nondairy ingredients (≤30% w/w) (Brasil 2007). Moisture content decreased in liposome‐enriched samples (83.77–83.93 g/100 g) versus the control (86.22 g/100 g), likely due to added solids from lyophilized liposomes. Protein content also dropped (3.71–3.79 g/100 g) compared to the control (4.13 g/100 g), mainly due to the lipid‐rich composition of liposomes. This reduction is attributed to a dilution effect rather than protein degradation, as lipid‐rich liposome dispersion partially replaced the protein fraction of the yogurt matrix. In absolute terms, the decrease was limited (approximately 0.3–0.4 g/100 g) and remained within the typical protein range reported for whole milk yogurts. Lipid content increased, especially in the P90G formulation (4.28 g/100 g), while coated liposomes showed lower lipid levels, likely due to pectin dilution. Ash content was higher in coated samples, potentially due to minerals from apple‐derived pectin. Carbohydrates increased in all treated samples, reflecting sucrose (cryoprotectant) and pectin contributions, with YR2 showing the highest value (8.13 g/100 g). These shifts reflect expected effects from incorporating lyophilized vesicles. From a nutritional perspective, although statistically significant increases in lipid and carbohydrate contents were observed, the overall macronutrient profile of the fortified yogurts remained comparable to that of conventional whole milk yogurts and did not result in an excessive caloric density per serving. Importantly, the objective of the present formulation was nutritional fortification through the delivery of VD3 and VB12 rather than the development of a therapeutic or high‐energy product. Accordingly, the fortified yogurts are intended for general consumption, maintaining a nutritional profile consistent with that of commercially available whole milk yogurts.

**TABLE 4:** Proximate composition of yogurt formulations enriched with lyophilized liposomes coencapsulating vitamins D_3_ and B_12_. blank (unfortified), Y90G (containing liposomes produced with Phospholipon 90G), YS45 (containing liposomes produced with Lipoid S45), YR1 (containing pectin‐coated LS45‐R1 liposomes), and YR2 (containing pectin‐coated LS45‐R2 liposomes). R1 and R2 correspond to the addition of 2.5 and 5.0 mg/mL pectin, respectively, during liposome production.

	Components (g/100 g of yogurts)					
	Moisture	Proteins	Lipids	Ash	Fibers	Carbohydrates
Blank	86.2^A^ ± 0.01	4.13^A^ ± 0.03	2.54^E^ ± 0.04	0.86^C^ ± 0.00	0.22^A^ ± 0.03	6.04^E^ ± 0.00
Y90G	83.9^B^ ± 0.00	3.75^B^ ± 0.02	4.28^A^ ± 0.06	0.86^C^ ± 0.01	0.03^BC^ ± 0.00	7.15^D^ ± 0.04
YS45	83.8^E^ ± 0.00	3.71^B^ ± 0.03	3.96^B^ ± 0.02	0.85^C^ ± 0.01	0.01^C^ ± 0.00	7.71^C^ ± 0.01
YR1	83.8^D^ ± 0.00	3.79^B^ ± 0.00	3.25^C^ ± 0.02	1.31^A^ ± 0.00	0.05^B^ ± 0.01	7.81^B^ ± 0.03
YR2	83.9^C^ ± 0.01	3.76^B^ ± 0.07	2.94^D^ ± 0.01	1.26^B^ ± 0.00	0.04^BC^ ± 0.00	8.13^A^ ± 0.05

*Note*: Averages followed by the same capital letter in the same column were not significantly different (*p* > 0.05) according to Tukey's test.

#### 3.3.2 Physicochemical Stability

Table 5 summarizes the physicochemical data collected on Days 1, 15, and 30. All samples had *A*_w_ around 0.99, typical for yogurt, with no significant changes during storage (Chaves et al. 2021). The control consistently showed the lowest pH (4.0), with no variation, aligning with commercial stable yogurt profiles (Food and Drug Administration 2023). Noncoated liposome samples maintained a stable pH of 4.1. Pectin‐coated liposome samples had higher initial pH (4.62 and 4.55), which slightly decreased by Day 15 (to 4.54 and 4.47) but remained above other formulations, likely due to pectin's buffering capacity (Ferreira et al. 2025). Titratable acidity ranged from 1.00 to 1.29 g lactic acid/100 g on Day 1, within legal limits (Brasil 2007). Syneresis varied: YS45 showed the highest (34.3 g whey/100 g yogurt), followed by Y90G and the control. Coated‐containing yogurts, especially YR2 (22.0 g whey/100 g yogurt), exhibited reduced syneresis, likely due to pectin's gelling and anionic properties, which enhance water retention by reinforcing the casein network (Arab et al. 2023). Overall, lyophilized liposomes, particularly pectin‐coated, improved yogurt stability by reducing whey separation while maintaining appropriate acidity, supporting their use in functional dairy applications.

**TABLE 5:** Physicochemical properties of yogurt formulations enriched with lyophilized liposomes coencapsulating vitamins D_3_ and B_12_, evaluated over 1, 15, and 30 days of refrigerated storage. Blank (unfortified), Y90G (containing liposomes produced with Phospholipon 90G), YS45 (containing liposomes produced with Lipoid S45), YR1 (containing pectin‐coated LS45‐R1 liposomes), and YR2 (containing pectin‐coated LS45‐R2 liposomes). R1 and R2 correspond to the addition of 2.5 and 5.0 mg/mL pectin, respectively, during liposome production.

Storage time (days)	Formulation	*A*_w_	pH	Titratable acidity (g lactic acid/100 g yogurt)	Syneresis (g exudate/100 g yogurt)
1	Blank	0.993^A,a^ ± 0.002	4.03^B,a^ ± 0.05	1.10^B,b^ ± 0.01	30.0^B,a^ ± 2.13
	Y90G	0.986^B,a^ ± 0.001	4.14^B,a^ ± 0.04	1.10^B,b^ ± 0.02	31.4^B,a^ ± 1.34
	YS45	0.988^AB,a^ ± 0.002	4.15^B,a^ ± 0.05	1.00^C,b^ ± 0.01	34.3^A,a^ ± 0.89
	YR1	0.985^B,a^ ± 0.002	4.62^A,a^ ± 0.01	1.18^A,c^ ± 0.01	26.3^C,a^ ± 2.90
	YR2	0.984^B,a^ ± 0.002	4.55^A,a^ ± 0.02	1.19^A,b^ ± 0.02	22.0^D,a^ ± 1.81
15	Blank	0.987^A,b^ ± 0.003	4.00^C,a^ ± 0.03	1.11^B,ab^ ± 0.02	30.9^A,a^ ± 1.66
	Y90G	0.985^A,a^ ± 0.003	4.06^BC,a^ ± 0.07	1.09^B,b^ ± 0.02	30.9^A,a^ ± 1.87
	YS45	0.986^A,a^ ± 0.003	4.14^B,a^ ± 0.02	1.05^C,a^ ± 0.03	30.8^A,a^ ± 2.15
	YR1	0.991^A,a^ ± 0.004	4.54^A,b^ ± 0.01	1.29^A,a^ ± 0.04	25.0^B,a^ ± 2.12
	YR2	0.984^A,a^ ± 0.002	4.47^A,b^ ± 0.02	1.28^A,a^ ± 0.04	24.5^B,a^ ± 2.66
30	Blank	0.991^A,ab^ ± 0.002	4.04^D,a^ ± 0.01	1.12^B,a^ ± 0.01	29.9^A,a^ ± 1.54
	Y90G	0.987^A,a^ ± 0.002	4.15^C,a^ ± 0.02	1.13^B,a^ ± 0.01	31.1^A,a^ ± 1.58
	YS45	0.987^A,a^ ± 0.004	4.14^C,a^ ± 0.03	1.03^C,a^ ± 0.01	29.5^A,a^ ± 1.35
	YR1	0.989^A,a^ ± 0.005	4.54^A,b^ ± 0.00	1.24^A,b^ ± 0.01	26.0^B,a^ ± 1.65
	YR2	0.988^A,a^ ± 0.003	4.46^B,b^ ± 0.01	1.25^A,a^ ± 0.01	23.8^B,a^ ± 2.31

*Note*: Averages followed by different capital letters are significantly different (*p* < 0.05) for samples with different formulations on the same day of storage. Averages followed by different lowercase letters are significantly different (*p* < 0.05) for samples with the same formulation on different storage days.

#### 3.3.3 Colorimetric Parameters and Vitamin Retention

To evaluate vitamin stability during storage, yogurt formulations were assessed for vitamin content and color. VB12's pink hue allowed indirect monitoring via instrumental colorimetry. As shown in Table 6, lyophilized liposome addition significantly altered yogurt appearance. Compared to the control (typical white–yellow hue), formulations with encapsulated vitamins showed higher a* values, indicating a pink shift, especially in uncoated samples (7.68 and 7.54 on Day 1). Coated‐containing yogurts had lower a* values (4.93 and 4.51), producing a subtler hue, likely due to the noticeably weaker pink color attributed to pectin‐coated lyophilized liposomes (Figure 2). C*_ab_ and h*_ab_ values also decreased, indicating a pink but less saturated tone. These values, along with low Δ*E* (<0.55), remained stable over 30 days, confirming good color retention and VB12 stability.

**TABLE 6:** Colorimetric parameters and vitamin concentrations in yogurt formulations evaluated on Days 1 and 30 of refrigerated storage. Blank (unfortified), Y90G (containing liposomes produced with Phospholipon 90G), YS45 (containing liposomes produced with Lipoid S45), YR1 (containing pectin‐coated LS45‐R1 liposomes), and YR2 (containing pectin‐coated LS45‐R2 liposomes). R1 and R2 correspond to the addition of 2.5 and 5.0 mg/mL pectin, respectively, during liposome production.

		Formulation									
		Blank		Y90G		YS45		YR1		YR2	
Storage day		1	30	1	30	1	30	1	30	1	30
Colorimetric parameters	L*	96.5^A,a^ ± 0.19	96.6^A,a^ ± 0.24	90.4^A,c^ ± 0.10	90.2^A,c^ ± 0.05	89.6^A,d^ ± 0.18	90.0^A,c^ ± 0.04	91.0^B,b^ ± 0.03	91.4^A,b^ ± 0.09	91.0^A,b^ ± 0.08	91.3^A,b^ ± 0.18
	a*	−1.65^A,d^ ± 0.08	−1.59^A,e^ ± 0.05	7.68^B,a^ ± 0.09	8.13^A,a^ ± 0.08	7.54^A,a^ ± 0.11	7.78^A,b^ ± 0.11	4.93^B,b^ ± 0.08	5.28^A,c^ ± 0.09	4.51^A,c^ ± 0.10	4.75^A,d^ ± 0.08
	b*	11.2^A,a^ ± 0.02	11.1^A,a^ ± 0.10	4.00^B,e^ ± 0.05	4.17^A,e^ ± 0.03	5.68^A,d^ ± 0.08	5.85^A,d^ ± 0.07	7.38^A,c^ ± 0.06	7.50^A,c^ ± 0.05	8.08^A,b^ ± 0.07	8.26^A,b^ ± 0.10
	C*	11.3^A,a^ ± 0.03	11.2^A,a^ ± 0.10	8.66^B,c^ ± 0.10	9.14^A,c^ ± 0.09	9.44^A,b^ ± 0.11	9.73^A,b^ ± 0.13	8.88^B,c^ ± 0.03	9.17^A,c^ ± 0.04	9.26^B,b^ ± 0.06	9.53^A,b^ ± 0.06
	h*	98.4^A,a^ ± 0.39	98.2^A,a^ ± 0.17	27.5^A,e^ ± 0.18	27.2^A,e^ ± 0.08	37.0^A,d^ ± 0.41	37.0^A,d^ ± 0.19	56.3^A,c^ ± 0.61	54.8^B,c^ ± 0.27	60.9^A,b^ ± 0.67	60.1^A,b^ ± 0.67
	Δ*E*	0.00^B,a^ ± 0.00	0.38^A,a^ ± 0.17	0.00^B,a^ ± 0.00	0.51^A,a^ ± 0.09	0.00^A,a^ ± 0.00	0.45^A,a^ ± 0.24	0.00^B,a^ ± 0.00	0.54^A,a^ ± 0.10	0.00^B,a^ ± 0.00	0.47^A,a^ ± 0.10
Vitamin content	VD3 (IU/g yogurt)	—	—	583^A^ ± 20.6	632^A^ ± 33.7	137^A^ ± 8.80	110^B^ ± 4.82	99.0^A^ ± 2.10	84.8^B^ ± 4.78	93.3^A^ ± 1.73	76.5^B^ ± 1.27
	VB12 (µg/g yogurt)	—	—	18.3^A^ ± 0.14	18.1^A^ ± 0.02	16.9^A^ ± 0.23	16.6^A^ ± 0.09	9.83^A^ ± 0.14	10.1^A^ ± 0.34	9.23^A^ ± 0.37	8.78^A^ ± 0.33
Theoretical vitamin concentration	VD3 (IU/g yogurt)	—	—	2667 ± 10	—	2667 ± 10	—	2286 ± 10	—	2000 ± 10	—
	VB12 (µg/g yogurt)	—	—	27 ± 1	—	27 ± 1	—	23 ± 1	—	20 ± 1	—

*Note*: Averages followed by different lowercase letters are significantly different (*p* < 0.05) for samples with different formulations on the same day of storage. Averages followed by different capital letters are significantly different (*p* < 0.05) for samples with the same formulation on different storage days.

Vitamin concentrations were lower than theoretical values (Table 6), likely due to analytical challenges. VD3's lipophilic nature and matrix interferences (from yogurt proteins, phospholipids, sucrose, pectin) may have hindered extraction and quantification, despite using methanol‐based methods (Yin et al. 2019). Still, the P90G‐based yogurt showed the highest vitamin levels after 30 days (632 IU VD3 and 18.1 µg VB12), suggesting strong protective effects. YS45 and coated samples had lower VD3 recovery, possibly due to stronger lipophilic interactions or matrix entrapment. However, vitamin levels remained stable post‐storage, highlighting the effectiveness of liposomal encapsulation.

#### 3.3.4 Microbiological Characterization

Yogurt formulations were tested for microbial quality over 30 days of refrigerated storage, with evaluations every 15 days. No filamentous fungi, yeasts, *E. coli*, or coliforms were detected in any samples, confirming microbiological safety and hygienic production. Table S2 shows LAB counts across formulations. All samples maintained viable LAB above the legal threshold of 10^7^ CFU/mL (Brasil 2007; Codex Alimentarius 2018). The control started at 1.04 × 10^8^ CFU/mL, declining to 3.23 × 10^7^ CFU/mL by Day 30. Liposome‐containing samples showed more stable LAB levels, with Y90G maintaining the highest at 4.72 × 10^7^ CFU/mL. YS45 and YR2 also retained consistent counts, with YS45 showing an increase from Day 1 to 15. These findings suggest liposome incorporation did not harm LAB viability and may have supported microbial stability in the yogurt matrix.

#### 3.3.5 Sensory Evaluation

**FIGURE 4:**
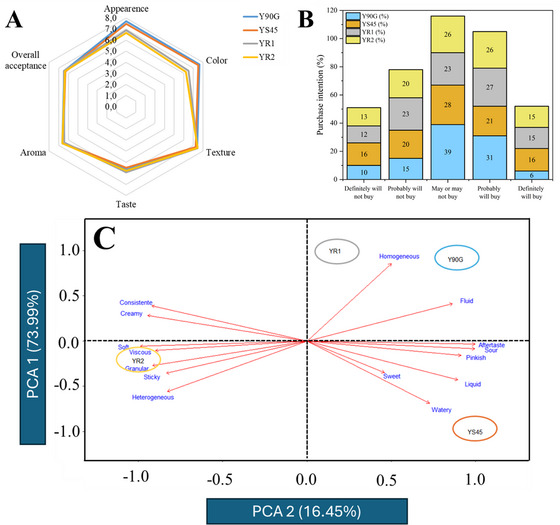
Sensory analysis of yogurt formulations. (A) Radar plot showing sensory acceptance scores for attributes evaluated for yogurt formulations enriched with lyophilized liposomes coencapsulating vitamins D_3_ and B_12_; (B) purchase intention and (C) principal component analysis (PCA) of descriptive sensory attributes, showing the distribution of formulations on the basis of panelist perceptions. Samples: Y90G (containing liposomes produced with Phospholipon 90G), YS45 (containing liposomes produced with Lipoid S45), YR1 (containing pectin‐coated LS45‐R1 liposomes), and YR2 (containing pectin‐coated LS45‐R2 liposomes). R1 and R2 correspond to the addition of 2.5 and 5.0 mg/mL pectin, respectively, during liposome production.

Yogurt samples were evaluated using affective testing and CATA method. Affective tests assessed *appearance*, *color*, *texture*, *taste*, *aroma*, *overall acceptance*, and *purchase intention*, while the CATA method captured consumer perceptions via descriptive terms. All evaluations occurred 24 h post‐production after storage at 10°C. Figure 4A shows the affective test results. Appearance and color differed significantly (*p* < 0.05) between yogurts with uncoated versus coated liposomes, attributed to the more intense pink color in uncoated formulations, consistent with instrumental color data and the presence of VB12, as no artificial coloring was used. Other sensory attributes showed no significant differences (*p* > 0.05). All samples received purchase intention scores of ∼3 on a 5‐point scale (“may or may not buy”) and overall acceptance scores of ∼6 on a 9‐point scale (“*liked slightly*” to “*liked moderately*”), indicating favorable perception despite no added sweeteners, flavoring, or aromas (Figure 4B). Results indicate liposomes did not negatively affect sensory appeal. Notably, no difference in perception was found between purified (P90G) and unpurified (LS45) phospholipids, suggesting unpurified sources could reduce production costs without compromising acceptability. It is important to observe that purchase intention was evaluated solely based on sensory perception, without providing consumers with any price or cost‐related information. Therefore, the cost implications discussed here are derived from a technological and ingredient sourcing perspective rather than from consumer economic perception. The use of unpurified phospholipids represents a well‐documented strategy to reduce raw material costs, and the present results indicate that this substitution does not compromise sensory acceptance.

CATA results showed that *homogeneous*, *sour*, and *creamy* were the most cited descriptors (>50%). *Aftertaste* was cited by 20%–30% of consumers, which may be associated with the presence of phospholipids and pectin in the formulations. *Pinkish* was noted by 80% (Y90G) and 78% (YS45), but only ∼50% for YR1 and YR2, with statistically significant differences among samples confirmed by Cochran's *Q* test followed by McNemar's post hoc comparisons (*p* < 0.05), aligning with affective test scores. As expected for unsweetened yogurt, *sour* was cited by 60%, and taste received moderate (∼5–6) hedonic scores, suggesting some reduced preference, but with promising purchase intention. CATA term frequencies were first statistically evaluated using Cochran's *Q* test (*p* < 0.05), followed by McNemar's test for pairwise comparisons (Table 7), and subsequently analyzed using principal component analysis (PCA; Figure 4C) to visualize relationships between samples and sensory descriptors. Y90G and YR1 showed similar sensory profiles and were primarily associated with homogeneous and fluid attributes, consistent with their higher frequencies for these descriptors and the absence of significant differences between them for several texture‐related terms. YS45 appeared nearby in the PCA space and was more closely associated with watery and liquid descriptors, which were cited significantly more frequently for this formulation (*p* < 0.05). In contrast, YR2 was clearly separated in a different quadrant and was associated with viscous, granular, sticky, heterogeneous, and consistent descriptors, all of which showed significantly higher citation frequencies compared to Y90G and YS45 (*p* < 0.05). Despite identical liposome concentrations, YR2's higher pectin content altered texture perception, increasing viscosity and reducing homogeneity. YR1, with less coating, retained a more uniform appearance and intermediate texture perception, explaining its proximity to Y90G in the PCA.

**TABLE 7:** Number (percentage, %) of consumers who selected each term from the CATA (check‐all‐that‐apply) method to describe the different yogurt formulations.

	Yogurt formulations			
Attributes	Y90G	YS45	YR1	YR2
Sweet	14 (11.4%)^b^	28 (22.8%)^a^	25 (20.3%)^a^ ^b^	14 (11.4%)^b^
Fluid	54 (43.9%)^a^	42 (34.1%)^a^ ^b^	40 (32.5%)^b^	28 (22.8%)^c^
Viscous	30 (24.4%)^b^	35 (28.5%)^a^ ^b^	42 (34.1%)^a^ ^b^	46 (37.4%)^a^
Watery	8 (6.5%)^a^ ^b^	14 (11.4%)^a^	5 (4.1%)^b^	5 (4.1%)^b^
Creamy	86 (69.9%)^b^	82 (66.7%)^b^	96 (78.0%)^a^ ^b^	101 (82.1%)^a^
Liquid	13 (10.6%)^a^ ^b^	23 (18.7%)^a^	9 (7.3%)^b^	2 (1.6%)^c^
Consistent	60 (48.8%)^b^	49 (39.8%)^c^	71 (57.7%)^a^ ^b^	79 (64.2%)^a^

*Note*: Means followed by different lowercase letters differ statistically from each other according to Cochran's *Q* test (*p* < 0.05), followed by the post hoc test. McNemar's hoc.

#### 3.3.6 Rheological Characterization

**FIGURE 5:**
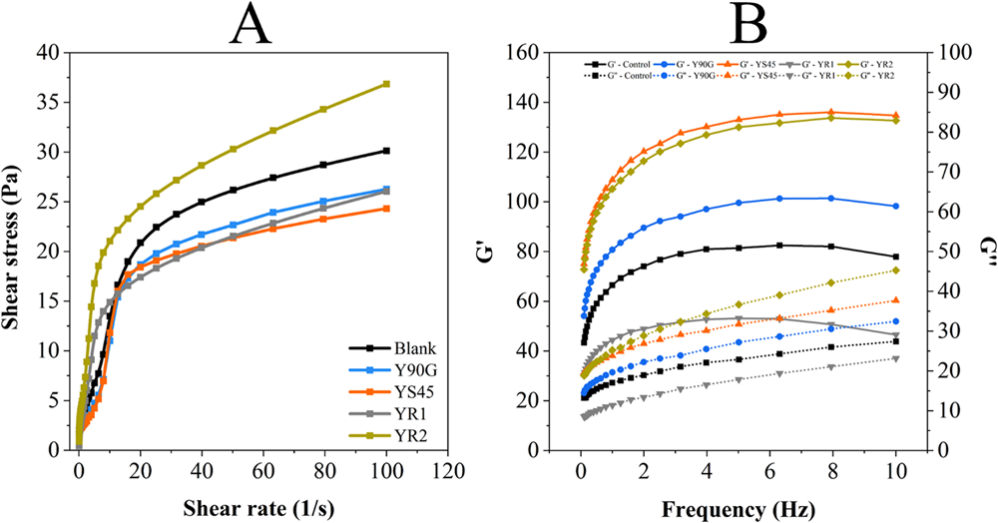
Rheological properties of yogurt samples enriched with lyophilized liposomes coencapsulating vitamin D_3_ and B_12_. (A) Flow behavior curves showing shear stress (Pa) as a function of shear rate (1/s) and (B) frequency sweep analysis depicting the storage modulus (*G*′, black symbols) and loss modulus (*G*″, red symbols) as a function of frequency (Hz). Blank (unfortified), Y90G (containing liposomes produced with Phospholipon 90G), YS45 (containing liposomes produced with Lipoid S45), YR1 (containing pectin‐coated LS45‐R1 liposomes), and YR2 (containing pectin‐coated LS45‐R2 liposomes). R1 and R2 correspond to the addition of 2.5 and 5.0 mg/mL pectin, respectively, during liposome production.

The rheological behavior of yogurt samples was assessed via flow curve analysis and dynamic oscillatory testing. Flow curves (Figure 5A) were fitted to the Herschel–Bulkley model, suitable for describing the non‐Newtonian flow of semisolid foods such as yogurt (Brito‐Oliveira et al. 2024; Chaves et al. 2021), with parameters in Table S3. All samples showed flow behavior index (*n*) values below 1, confirming pseudoplastic behavior typical of fermented dairy products. This results from the alignment of protein and polysaccharide structures under shear, enabling easier flow. Significant differences were found in the consistency coefficient (*K*), with the highest values in YR2 (11.59 ± 0.742 Pa s*^n^*) and YR1 (8.249 ± 0.951 Pa s*^n^*), both containing pectin‐coated liposomes. Pectin likely interacts with milk proteins, enhancing gel strength. In contrast, the control and samples with uncoated liposomes showed lower *K* values (∼4.1–4.5 Pa s*^n^*), indicating little structural impact.

Yield stress (*σ*_0_), indicating the stress needed to start flow, was significantly higher in yogurts with coated liposomes, reflecting a more robust gel network. Near‐zero or negative σ_0_ values in the control, Y90G, and YS45 suggest weaker structures. Apparent viscosity followed similar trends, with YR2 highest (1.76 ± 0.04 Pa s), while other samples ranged from 1.22 to 1.32 Pa s without significant differences. YR2 also had the most pronounced thixotropic behavior (−289.2 ± 20.65 Pa/s), reflecting substantial structure break‐down and slow recovery, likely due to the denser network formed by pectin.

Oscillatory tests (Figure 5B) showed all samples had elastic modulus (*G*′) > viscous modulus (*G*″), confirming gel‐like properties. Incorporation of pectin‐coated liposomes, especially in YR2, significantly altered the yogurt's rheological profile. Increases in viscosity, yield stress, and consistency coefficient, along with stronger thixotropy, suggest enhanced gel structure via biopolymer‐protein interactions. Notably, YR2 was linked to creamy and viscous descriptors in the CATA test, reflecting its rheological strength. In contrast, formulations without pectin were associated with *liquid* and *watery*, indicating weaker structure. These findings suggest that pectin not only strengthens the yogurt matrix but also enhances sensory attributes such as creaminess and texture.

#### 3.3.7 Static In Vitro Digestion of Yogurt: Vitamin Release and Protein Hydrolysis

**FIGURE 6:**
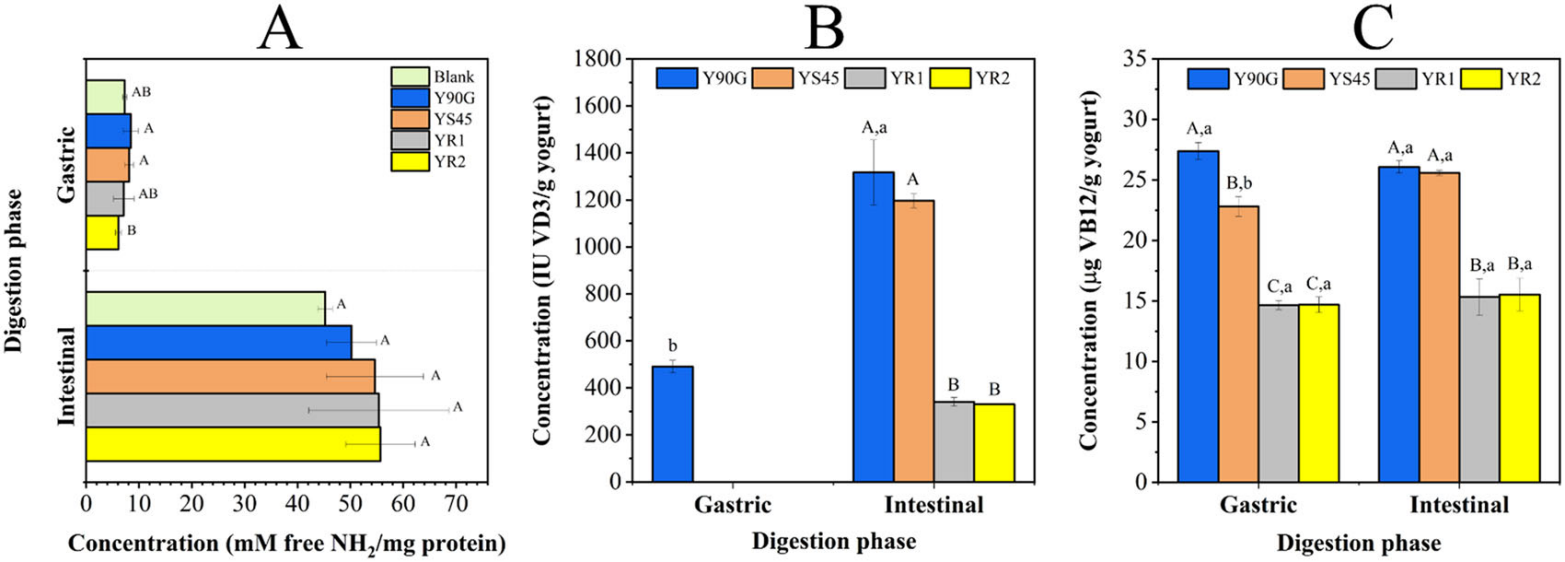
Effect of liposome encapsulation on protein hydrolysis and vitamin release during static in vitro digestion of yogurt. (A) Degree of protein hydrolysis in the gastric and intestinal phases; Concentrations of (B) vitamin D_3_ (IU/g yogurt) and (C) vitamin B_12_ (µg/g yogurt) in the gastric and intestinal phases. Blank (unfortified), Y90G (containing liposomes produced with Phospholipon 90G), YS45 (containing liposomes produced with Lipoid S45), YR1 (containing pectin‐coated LS45‐R1 liposomes), and YR2 (containing pectin‐coated LS45‐R2 liposomes). R1 and R2 correspond to the addition of 2.5 and 5.0 mg/mL pectin, respectively, during liposome production. Means followed by the same uppercase letter represent nonsignificant differences (*p* < 0.05) between yogurt formulations at the same stage of digestion. Means followed by the same lowercase letter represent nonsignificant differences (*p* < 0.05) between the same yogurt formulations at different stages of in vitro digestion.

Figure 6A shows proteolysis levels during digestion, measured by free amine group concentration (mM NH_2_/mg of protein). In the gastric phase, uncoated liposome formulations showed slightly higher protein hydrolysis (8.5 and 8.1 mM NH_2_/mg of protein) than the control yogurt (7.3 mM NH_2_/mg of protein) though not significantly. In contrast, YR2, containing a high concentration of pectin‐coated liposomes, had the lowest hydrolysis (6.1 mM NH_2_/mg of protein), significantly differing from most formulations. This aligns with the rheological results suggesting that coated liposomes reinforced the yogurt matrix, possibly limiting pepsin access by forming protein–polysaccharide interactions. Conversely, uncoated liposomes likely allowed greater enzyme accessibility.

Figure 6B,C show vitamin bioaccessibility. VD3 was detectable during the gastric phase only in Y90G, while other formulations released it only after intestinal digestion, a beneficial outcome given VD3 absorption occurs mainly in the small intestine. This may result from VD3‐partially hydrolyzed milk protein interactions in the stomach that protect the vitamin until release. These observations align with the findings of Ghiraldi et al. (2024), who reported increased VD3 bioaccessibility in whey protein isolate‐based gels. The authors attributed this to the formation of β‐lactoglobulin–VD3 complexes, which are resistant to both gastric and intestinal enzymatic degradation. However, VD3 bioaccessibility was lower in pectin‐coated formulations, suggesting that although the coating protected liposome in the stomach, it may have limited their disintegration in the intestine. VB12 levels remained stable during digestion, except for YS45. This is consistent with VB12's natural absorption pathway involving haptocorrin, a salivary glycoprotein, and then to intrinsic factor (IF), which is secreted by gastric parietal cells. The VB12–IF complex is critical for absorption in the ileum via receptor‐mediated endocytosis (Guéant et al. 2022).

The distinct behavior of VD3 during the gastric and intestinal phases can be also mainly attributed to pH‐dependent effects on both the food matrix and the liposomal carrier. Recent evidence indicated that gastric pH plays a critical role in VD3 stability and bioaccessibility, as variations in acidity can modulate vitamin retention, release, and even degradation prior to the intestinal phase (Pasidi and Vareltzis 2024). During the gastric phase (pH ∼2–3), the low aqueous solubility of VD3, along with protein aggregation near the isoelectric point of caseins, probably favored its retention within protein‐lipid or protein‐vitamin complexes, thereby limiting its release. Under these acidic conditions, partial protonation of liposomal surfaces may further reduce membrane permeability, contributing to vitamin protection in the stomach. Upon transition to the intestinal phase (pH ∼6.5–7.5), increased pH, bile salts, and pancreatic enzymes promote liposome destabilization, lipid digestion, and mixed micelle formation, which are essential steps for VD3 solubilization and absorption. Indeed, mixed micelle formation in the small intestine is a prerequisite for the uptake of lipophilic vitamins such as VD3, and delivery systems that enhance micellar incorporation have consistently been shown to improve intestinal bioaccessibility (Mulrooney et al. 2021, 2022). In this context, VD3 associated with lipid‐based or protein‐based complexes, including dairy protein carriers, has been reported to exhibit enhanced bioaccessibility, as these structures protect the vitamin in the gastric environment while facilitating its release and micellization in the intestine (Cohen et al. 2021). Therefore, the absence or delayed detection of VD3 during the gastric phase observed for most formulations in this study may be considered advantageous, as premature release under acidic conditions could compromise vitamin stability and absorption efficiency. Conversely, the lower intestinal bioaccessibility observed for pectin‐coated liposomes suggests that, although polysaccharide coatings enhance gastric stability, they may interfere in the lipid digestion and mixed micelle formation at intestinal pH, likely due to reduced enzymatic accessibility or interactions with digestion products (Espinal‐Ruiz et al. 2014; Tan et al. 2020). Similar trade‐offs between gastric protection and intestinal release have been reported for polysaccharide‐coated lipid carriers, highlighting the need to balance structural stability with digestibility to optimize VD3 absorption (Tan et al. 2019).

According to Brazilian legislation, the recommended daily intake (RDI) for adults is 5 µg (200 IU) of VD and 2.4 µg of VB12 (Brazil 2005). The Institute of Medicine (IOM 2011) recommends a daily intake of 600 IU of VD for adults aged 18–70 years and 800 IU for individuals over 70 years. The tolerable upper intake level is established at 4000 IU/day (IOM 2011). Within this context, even small portions of the formulated yogurt could provide vitamin levels consistent with the RDI, making them a promising dietary source and a potential alternative to conventional supplementation practices.

## 4 Conclusion

This study demonstrated the feasibility of using lyophilized liposomes to enrich yogurt with vitamins D_3_ and B_12_. The liposomes were stable carriers, with better vitamin retention observed in formulations using nonhydrogenated phospholipids, regardless of pectin coating. Once incorporated into yogurt, they effectively delivered functional benefits without compromising quality. VB12 added pinkish hue, enhancing visual appeal while retaining bioactivity during digestion. In vitro analysis confirmed the yogurt could supply physiologically relevant amounts of both vitamins, supporting their nutritional contribution. Pectin‐coated liposomes improved VD3 retention during digestion, indicating a protective effect. Sensory evaluation showed high consumer acceptance, with significantly more positive than negative responses. Overall, lyophilized liposomes offer a promising strategy for delivering essential micronutrients in functional dairy products, ensuring stability, nutritional efficacy, and consumer appeal.

## Author Contributions

**Letícia S. Ferreira**: conceptualization, investigation, writing – original draft, methodology, validation, visualization, writing – review and editing, software, formal analysis, data curation, project administration. **Eduarda Habermann Luvizzotti**: investigation, methodology, formal analysis. **Marluci Ghiraldi**: conceptualization, investigation, methodology. **Matheus Andrade Chaves**: methodology, writing – review and editing, data curation, supervision. **Samantha C. Pinho**: data curation, supervision, resources, funding acquisition, writing – original draft, methodology, conceptualization.

## Conflicts of Interest

The authors declare no conflicts of interest.

## Supporting information




**Supplementary Materials**: jfds70870‐sup‐0001‐SuppMat.docx
