# Supplementary material for: Yogurt Fortification With Lyophilized Liposomes Coencapsulating Vitamins D3 and B12: Physicochemical Characterization, Sensory Evaluation and Static In Vitro Digestion
Source: J Food Sci. 2026 Feb 6;91(2):e70870. doi: 10.1111/1750-3841.70870 (PMC12877996; doi:10.1111/1750-3841.70870)
Supplement: Supplementary file 1 — Supplementary Materials: jfds70870‐sup‐0001‐SuppMat.docx [file JFDS-91-0-s001.docx]

**Yogurt fortification with lyophilized liposomes coencapsulating vitamins D_3_ and B_12_: physicochemical characterization, sensory evaluation and static in vitro digestion**

**Letícia S. Ferreira^1^, Eduarda Habermann Luvizzotti^1^, Marluci Ghiraldi^1^, Matheus Andrade Chaves^1,2^ and Samantha C. Pinho^1^***

^1^Laboratory of Encapsulation and Functional Foods (LEnAlis), Department of Food Engineering, School of Animal Science and Food Engineering (FZEA) - University of São Paulo (USP) - Av. Duque de Caxias Norte 225 – Jd Elite – Pirassununga – SP, Brazil 13635-900

^2^Laboratory of Molecular Morphophysiology and Development (LMMD), Department of Veterinary Medicine, School of Animal Science and Food Engineering (FZEA) - University of São Paulo (USP) - Av. Duque de Caxias Norte 225 – Jd Elite – Pirassununga – SP, Brazil 13635-900

***Supplementary material***

* Corresponding author: S. C. Pinho, Department of Food Engineering, School of Animal Science and Food Engineering (FZEA), University of São Paulo (USP), Av. Duque de Caxias Norte 225, Jd. Elite, Pirassununga, SP, Brazil 13635-900. Tel: +55-19-3565-4288.

*E-mail address:* samantha@usp.br

**Supplementary Table 1.** Colorimetric parameters a* and b* of lyophilized liposomes coencapsulating vitamins D_3_ and B_12_. Liposomes were produced with different food-grade phospholipids: Phospholipon 90G (P90G), Lipoid S45 (LS45), and pectin-coated formulations (LS45-R1 and LS45-R2). R1 and R2 correspond to an addition of 2.5 and 5.0 mg/mL of pectin during liposome production, respectively.

|  |  | Day 1 | Day 30 | Day 60 | Day 90 | Day 120 |
| --- | --- | --- | --- | --- | --- | --- |
| P90G | a* | 42.80^C^ ± 0.21 | 46.23^AB^ ± 0.09 | 46.41^A^ ± 0.01 | 45.74^B^ ± 0.24 | 45.90^AB^ ± 0.13 |
|  | b* | 5.38 ^B^ ± 0.07 | 7.89^A^ ± 0.04 | 8.08 ^A^ ± 0.01 | 7.92^A^ ± 0.23 | 8.18^A^ ± 0.05 |
| LS45 | a* | 44.44^B^ ± 0.01 | 46.92^A^ ± 0.20 | 46.77^A^ ± 0.02 | 43.38^C^ ± 0.06 | 43.52^C^ ± 0.10 |
|  | b* | 14.54^E^ ± 0.01 | 16.10^D^ ± 0.17 | 16.84^C^ ± 0.02 | 21.32^B^ ± 0.01 | 25.94^A^ ± 0.10 |
| LS45 – R1 | a* | 22.01^C^ ± 0.14 | 22.72^ABC^ ± 0.30 | 22.89^AB^ ± 0.08 | 23.47^A^ ± 0.42 | 22.39^BC^ ± 0.06 |
|  | b* | 9.06^C^ ± 0.04 | 9.31^BC^ ± 0.09 | 9.46^AB^ ± 0.04 | 9.60^A^ ± 0.15 | 9.28^BC^ ± 0.01 |
| LS45 – R2 | a* | 18.35^A^ ± 0.16 | 18.29^A^ ± 0.05 | 18.15^A^ ± 0.06 | 18.36^A^ ± 0.01 | 18.73^A^ ± 0.41 |
|  | b* | 11.28^A^ ± 0.09 | 11.18^A^ ± 0.04 | 11.12^A^ ± 0.03 | 11.21^A^ ± 0.01 | 11.41^A^ ± 0.25 |

Values are expressed as mean ± standard deviation (n = 3). Means within the same row followed by the same uppercase letter are not significantly different (p > 0.05), according to Tukey’s test.

**Supplementary Table 2.** Viable counts of lactic acid bacteria (LAB) in yogurt samples fortified with lyophilized liposomes coencapsulating vitamins D_3_ and B_12_, during 30 days of refrigerated storage. Blank (unfortified), Y90G (containing liposomes produced with Phospholipon 90G), YS45 (containing liposomes produced with Lipoid S45), YR1 (containing pectin-coated LS45-R1 liposomes), and YR2 (containing pectin-coated LS45-R2 liposomes). R1 and R2 correspond to an addition of 2.5 and 5.0 mg/mL of pectin during liposome production, respectively. Results are expressed as colony-forming units per gram (CFU/g).

|  | Storage day | | |
| --- | --- | --- | --- |
|  | 1 | 15 | 30 |
| Blank | 1.04^A,a^ x 10^8^ | 3.04^A,a^ x 10^7^ | 3.23^A,a^ x 10^7^ |
| Y90G | 4.51^A,ab^ x 10^7^ | 3.83^A,a^ x 10^7^ | 4.72^A,a^ x 10^7^ |
| YS45 | 2.35^A,b^ x 10^7^ | 4.63^A,a^ x 10^7^ | 4.72^A,a^ x 10^7^ |
| YR1 | 3.13^A,ab^ x 10^7^ | 2.68^A,a^ x 10^7^ | 2.58^A,a^ x 10^7^ |
| YR2 | 2.70^A,b^ x 10^7^ | 2.97^A,a^ x 10^7^ | 4.25^A,a^ x 10^7^ |

Values are expressed as mean ± standard deviation (n = 3). Lowercase letters indicate statistically significant differences (p < 0.05) among formulations on the same storage day. Uppercase letters indicate significant differences (p < 0.05) among storage days for the same formulations, according to Tukey’s test.

**Supplementary Table 3.** Rheological parameters of yogurt samples fortified with lyophilized liposomes coencapsulating vitamins D_3_ and B_12_ based on the Herschel-Bulkley model.

| Formulation | σ_0_ (Pa) | K’ (Pas) | n | R^2^ | Thixotropic (Pa/s) | Apparent viscosity (Pa.s) |
| --- | --- | --- | --- | --- | --- | --- |
| Blank | -0.193^A^ ± 0.070 | 4.515^C^ ± 0.275 | 0.423^A^ ± 0.004 | 62.83 | -125.9^AB^ ± 27.08 | 1.32^B^ ± 0.03 |
| Y90G | -0.250^A^ ± 0.043 | 4.103^C^ ± 0.008 | 0.426^A^ ± 0.012 | 79.29 | -18.69^A^ ± 0.226 | 1.22^B^ ± 0.02 |
| YS45 | -0.795^A^ ± 0.140 | 4.527^C^ ± 0.001 | 0.393^A^ ± 0.003 | 91.29 | -137.6^AB^ ± 72.84 | 1.27^B^ ± 0.01 |
| YR1 | -3.044^B^ ± 0.727 | 8.249^B^ ± 0.951 | 0.282^B^ ± 0.025 | 48.72 | -95.23^AB^ ± 75.34 | 1.25^B^ ± 0.01 |
| YR2 | -3.845^B^ ± 0.478 | 11.59^A^ ± 0.742 | 0.279^B^ ± 0.02 | 47.94 | -289.2^B^ ± 20.65 | 1.76^A^ ± 0.04 |

Values are expressed as mean ± standard deviation (n = 3). Means within the same column followed by the same uppercase letter are not significantly different (p > 0.05), according to Tukey’s test.
